# Frontotemporal dementia-associated N279K tau mutant disrupts subcellular vesicle trafficking and induces cellular stress in iPSC-derived neural stem cells

**DOI:** 10.1186/s13024-015-0042-7

**Published:** 2015-09-15

**Authors:** Melissa C. Wren, Jing Zhao, Chia-Chen Liu, Melissa E. Murray, Yuka Atagi, Mary D. Davis, Yuan Fu, Hirotaka J. Okano, Kotaro Ogaki, Audrey J. Strongosky, Pawel Tacik, Rosa Rademakers, Owen A. Ross, Dennis W. Dickson, Zbigniew K. Wszolek, Takahisa Kanekiyo, Guojun Bu

**Affiliations:** Department of Neuroscience, Mayo Clinic Jacksonville, 4500 San Pablo Road, Jacksonville, FL 32224 USA; Division of Regenerative Medicine, Jikei University School of Medicine, 3-25-8 Nishishinbashi, Minato-ku Tokyo, 105-8461 Japan; Department of Neurology, Mayo Clinic Jacksonville, 4500 San Pablo Road, Jacksonville, FL 32224 USA

**Keywords:** FTDP-17, iPSCs, N279K, Neural stem cells, PPND, Tau

## Abstract

**Background:**

Pallido-ponto-nigral degeneration (PPND), a major subtype of frontotemporal dementia with parkinsonism related to chromosome 17 (FTDP-17), is a progressive and terminal neurodegenerative disease caused by c.837 T > G mutation in the *MAPT* gene encoding microtubule-associated protein tau (rs63750756; N279K). This *MAPT* mutation induces alternative splicing of exon 10, resulting in a modification of microtubule-binding region of tau. Although mutations in the *MAPT* gene have been linked to multiple tauopathies including Alzheimer’s disease, frontotemporal dementia and progressive supranuclear palsy, knowledge regarding how tau N279K mutation causes PPND/FTDP-17 is limited.

**Results:**

We investigated the underlying disease mechanism associated with the N279K tau mutation using PPND/FTDP-17 patient-derived induced pluripotent stem cells (iPSCs) and autopsy brains. In iPSC-derived neural stem cells (NSCs), the N279K tau mutation induced an increased ratio of 4-repeat to 3-repeat tau and accumulation of stress granules indicating elevated cellular stress. More significant, NSCs derived from patients with the N279K tau mutation displayed impaired endocytic trafficking as evidenced by accumulation of endosomes and exosomes, and a reduction of lysosomes. Since there were no significant differences in cellular stress and distribution of subcellular organelles between control and N279K skin fibroblasts, N279K-related vesicle trafficking defects are likely specific to the neuronal lineage. Consistently, the levels of intracellular/luminal vesicle and exosome marker flotillin-1 were significantly increased in frontal and temporal cortices of PPND/FTDP-17 patients with the N279K tau mutation, events that were not seen in the occipital cortex which is the most spared cortical region in the patients.

**Conclusion:**

Together, our results demonstrate that alterations of intracellular vesicle trafficking in NSCs/neurons likely contribute to neurodegeneration as an important disease mechanism underlying the N279K tau mutation in PPND/FTDP-17.

## Background

Frontotemporal dementia with parkinsonism related to chromosome 17 (FTDP-17) prevails as one of the most common form of early-onset dementia [1]. FTDP-17 presents as a fulminant progressive neurodegenerative dementia, with no curative treatment or effective palliative relief. Clinically, FTDP-17 displays a triad of symptoms, with parkinsonism, behavioral changes and personality dysfunctions, and later cognitive impairments [1, 2]. Two genetic causes have been identified that lead to distinct forms of FTDP-17; mutations in progranulin (*GRN*) cause FTDP-17U (ubiquitin) with TDP-43 pathology and mutations in the microtubule-associated protein tau (*MAPT*) gene cause FTDP-17T (tau) [3]. Approximately one-half of all FTDP-17 cases are caused by autosomal dominant mutations in *MAPT.* The pallido-ponto-nigral degeneration (PPND) family is the largest and most comprehensively studied kindred of tau N279K mutant carriers and of all FTDP-17 families, containing to date, 59 affected individuals [4].

The N279K substitution is encoded within exon 10 of the *MAPT* gene locus and is one of the most frequent mutations in FTDP-17T patients [2]; together with mutations P301L and intron 10 + 16. Together these three mutations account for up to 60 % of FTDP-17T cases [5]. Multiple *MAPT* transcripts exist due to the alternative splicing of the *MAPT* gene, and transcripts of *MAPT* can be classified based on the inclusion/exclusion of exon 10 [6]. Most *MAPT* mutations including the N279K mutation increase the transcript containing exon 10 resulting in the overproduction of tau protein isoforms containing four tandem microtubule-binding domain repeats (4R-tau); whereas levels of the other major isoform in human brain containing only three repeats (3R-tau) is believed to be unaffected [7]. Although it is not fully understood how the increase in 4R-tau contributes to disease pathogenesis, *MAPT* mutations have been shown to cause multiple tauopathies [8–10]. It is believed that tauopathies are caused by aberrant hyperphosphorylation of tau, leading to the assembly of variable neurotoxic tau aggregates and deposition of insoluble tau fibers in both neurons and glia [11]. Progressive tau deposition in FTDP-17T patients is associated with severe neocortical atrophy of the frontal and temporal lobes, in association with the degeneration of medial temporal structures [12]. Destruction of the basal ganglia and depigmentation of the substantia nigra may also be present to some degree in *MAPT* mutant carriers, but are documented to be a consistent pathological hallmark in N279K kindreds [13, 14].

The exact mechanism owing to the cell death of specific neuronal populations in FTDP-17T remains to be identified; however, the characteristic dominant penetrance of the N279K tau mutant suggests a gain-of-toxic-function that results in specific subsets of degenerating cells. The induced pluripotent stem cell (iPSC) technology provides a novel and unparalleled approach to aid the study of molecular and cellular dynamics of disease pathogenesis by utilizing patient-specific cells with pathologically linked genetic mutations on the inherent genetic background [15], which has facilitated its exponential use as a novel tool to understand the pathogenesis of multiple neurodegenerative diseases [16]. Herein, we investigated the pathogenic mechanism underlying FTDP-17T with mutant N279K tau by using cells derived from two patients from the PPND family who are both clinically affected. Neural stem cells (NSCs) derived from the patient-specific iPSCs revealed abnormal amounts of cellular vesicle components in the presence of aberrantly spliced tau, accompanied by increases in several components of cellular stress. Correspondingly, altered cellular markers reflecting abnormal vesicle trafficking were observed in the autopsy brains of patients carrying the N279K mutation, supporting the results from the iPSC-based approach. Our findings demonstrate that patient-specific iPSCs can be used to model neurodegenerative diseases and uncover cellular dysfunctions associated with a disease-causing mutation.

## Results

### Generation and characterization of iPSCs

**Fig. 1:**
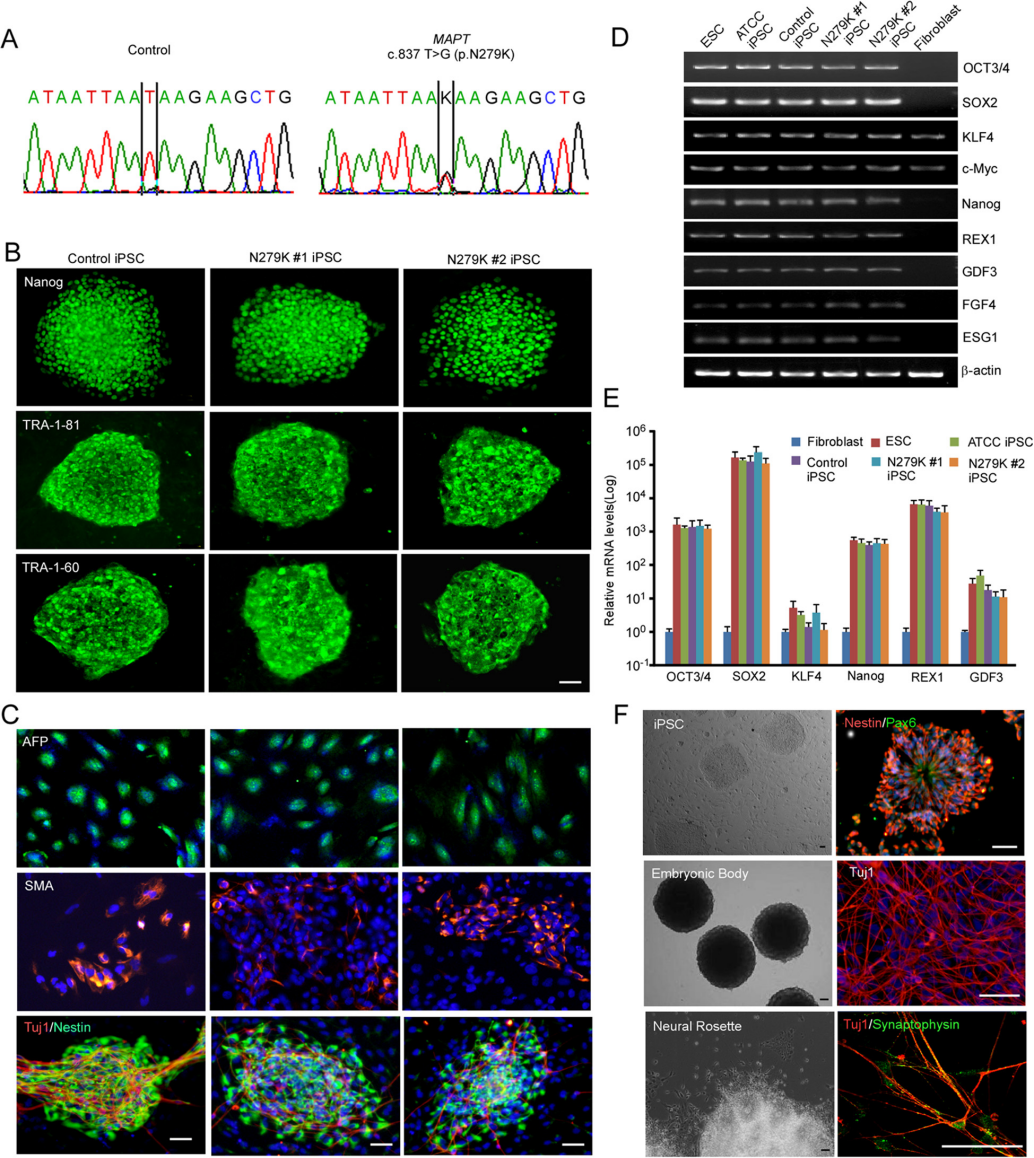
Generation and characterization of patient-specific iPSCs with N279K tau mutation. **a** Genomic DNA sequencing of the heterozygous *MAPT* missense mutation c.837 T > G (p.N279K) in fibroblasts from the PPND/FTDP-17 patients. **b** Immunostaining of pluripotency markers (Nanog, TRA-1-81 and TRA-1-60) in a control iPSC and two N279K iPSC lines. Scale bar, 50 μm. **c** *In vitro* differentiation of a control iPSC and two N279K iPSC lines into cells of all three germ layers. Cells were immunostained for AFP (*endoderm*), SMA (*mesoderm*), Tuj1/Nestin (*ectoderm*), and DAPI (*nucleus*). Scale bar, 50 μm. **d**-**e** Total mRNA levels of the reprogramming factors in a human ES cell line, a commercial iPSC line, a control iPSC line and two N279K iPSC lines relative to the values in a control fibroblast were assessed by qRT-PCR. Data are mean ± SEM from three independent experiments. **f** Phase-contrast images of the control iPSC line, embryonic bodies and neural rosettes. Immunostaining for the cells at different stages of neuronal differentiation; Nestin and Pax6 for NSCs, Tuj1 for mature neurons, synaptophysin for presynapses, and DAPI. Scale bar, 50 μm

Two PPND family members known as N279K tau mutant carriers were recruited into this study. Both were described as clinically affected at the time of dermal extraction. Skin biopsy specimens were immediately processed to generate primary fibroblasts. Commercially available human dermal fibroblasts, documented to be clinically normal, were used as a control. Genomic sequencing of exon 10 of the *MAPT* locus confirmed the retention of the c.837 T > G mutation (rs63750756; N279K) in fibroblasts from the PPND/FTDP-17T patients (Fig. 1a). The fibroblasts were transfected with three episomal vectors containing five transcription factors (OCT 3/4, SOX2, L-MYC, KLF4, LIN28) and p53 shRNA for the conversion to iPSCs, as previously described [17]. After 3-4 weeks of the transfection, approximately 30 iPSC colonies per subject were selected and expanded. We first confirmed that the iPSCs expressed several pluripotent stem cell specific markers, including Nanog, TRA-1-81 and TRA-1-60 by immunostaining (Fig. 1b). In addition, all iPSC lines could spontaneously differentiate into cell types of all three germ layers *in vitro* when they were stained for an endoderm marker α-fetoprotein (AFP), a mesoderm marker α-smooth muscle actin (SMA) and an ectoderm markers Tuj1 (β III tubulin) and Nestin (Fig. 1c).

We examined mRNA expression of pluripotent markers Oct3/4, Sox 2, Klf4, c-Myc, Nanog, REX, GDF3, FGF-4, and ESG1 by RT-PCR in the iPSC lines. While skin fibroblasts expressed very low levels of these mRNAs except Klf4 and c-Myc, all markers were up-regulated and/or induced in the iPSC lines, comparable to those of a human ES cell line (HUES2, Harvard Stem Cell Science) and a validated iPSC line (ATCC ACS-1019) (Fig. 1d, e). These results demonstrate the successful generation of patient-specific iPSC lines with N279K mutation, which have similar patterns of stem cell marker expression and the pluripotency along with the control iPSC. To confirm the differentiation methods of iPSC lines to neuronal linage, the control iPSC line was further directed to form embryonic bodies and converted to the neural lineage through the derivation of neurospheres to neural rosettes. Neural rosettes were selected and dissociated to produce Nestin-positive NSCs (Fig. 1f). NSCs derived from control iPSC line could successfully differentiate to Tuj1-positive mature neurons, where a presynaptic marker synaptophysin was also detected by immunostaining (Fig. 1f). However, in the case of NSCs from iPSC lines with mutant tau N279K, only a small percentage of NSCs differentiated into mature neurons, suggesting the mutation significantly affects neuronal survival. Those neurons from N279K iPSC lines that did survive the differentiation process rapidly degenerated, preventing their use in studying cellular functions. For these reasons, NSCs were used to study cellular dysfunctions associated with the mutant N279K tau.

### N279K tau mutation induces cellular stress in NSCs derived from patient-specific iPSCs

**Fig. 2:**
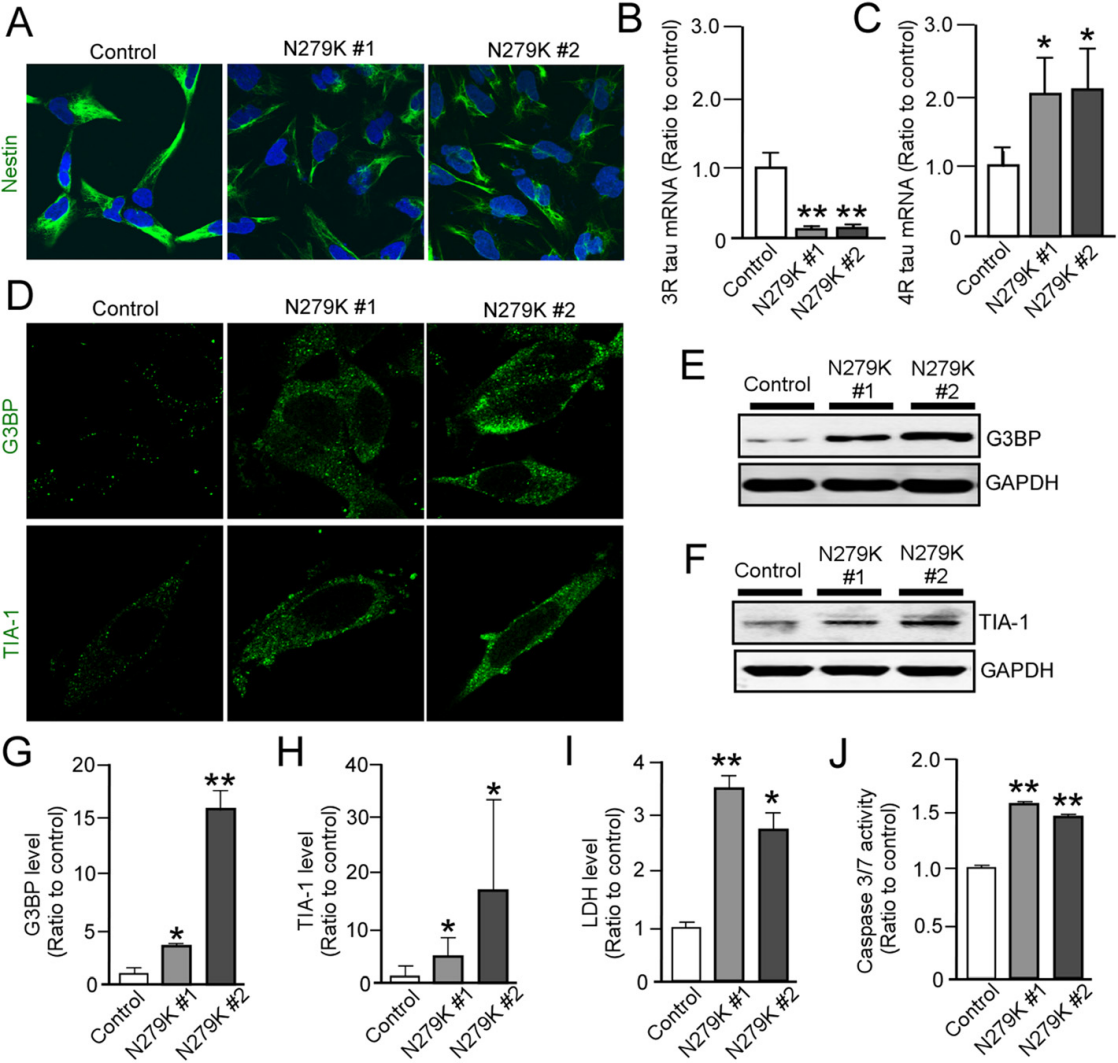
N279K neural stem cells exhibit altered tau isoform expression and enhanced cellular stress. **a** Immunostaining of NSC protein marker Nestin in a control and the patient-derived NSCs with N279K tau mutant. **b**, **c** Quantitative RT-PCR analysis of 3R-tau and 4R-tau mRNAs in control and N279K NSCs. Relative gene expression represents fold changes relative to that of control NSCs and normalized to β-actin expression. **d** Immunostaining of stress granule markers G3BP and TIA-1 in control and N279K NSCs. **e**-**h** Western blot analysis of stress granule proteins G3BP and TIA-1 in control and N279K NSCs. Quantification of Western blot densitometry of G3BP levels **g** and TIA-1 Levels **h**, normalized to GAPDH densitometry. **i** LDH quantification normalized to Bradford protein levels. **j** Caspase 3/7 activity was measured in control and N279K NSCs and the quantification was normalized to Bradford protein levels. Data are mean ± SEM from three independent experiments. *p < 0.05; **p < 0.01

To investigate potential effects of the N279K mutant tau in NSCs, we differentiated control iPSC and the PPND/FTDP-17T patient specific iPSC lines with N279K tau mutation to produce Nestin-positive NSCs (Fig. 2a). As expected, mRNA for human tau isoforms 3R-tau and 4R-tau were shown to be differentially expressed in mutants versus control NSCs. Levels of 3R-tau in N279K NSCs were significantly lower than the control NSCs (Fig. 2b), whereas 4R-tau mRNA levels were markedly increased in N279K NSCs (Fig. 2c). The mRNAs of both 3R-tau and 4R-tau were undetectable in the concomitant fibroblasts.

It has been recently reported that cellular stress induces aggregation of RNA-binding proteins resulting in the formation of inclusion bodies as stress granules [18], which are implicated in several neurodegenerative diseases including FTDP-17 [19]. Thus, we compared the qualitative frequency of stress granule puncta in control and N279K NSCs by immunostaining for G3BP and TIA-1. Confocal microscopy showed that N279K NSCs display a higher incidence of G3BP- or TIA-1-positive puncta than in control NSCs (Fig. 2d). Similarly, protein expression levels of G3BP (Fig. 2e, g) and TIA-1 (Fig. 2f, h) under basal conditions were higher in N279K NSCs than control NSCs when analyzed by Western blot. Consistent with these results, we also found increased lactate dehydrogenase (LDH) release into extracellular medium (Fig. 2i) and increased capase-3/7 activity in living cells from the N279K NSCs when compared to control NSCs (Fig. 2j). There were no significant differences between control and N279K skin fibroblasts in both LDH release (Fig. 4a) and capase-3/7 activity (Fig. 4b), suggesting that N279K-associated phenotypes are specific for the neuronal lineage. Taken together, these results indicate that the N279K tau mutation increases 4R-tau/3R-tau ratio and is associated with induced cellular stress as early as at the NSC stage.

### Intracellular vesicle components are altered in the NSCs with N279K tau mutation

**Fig. 3:**
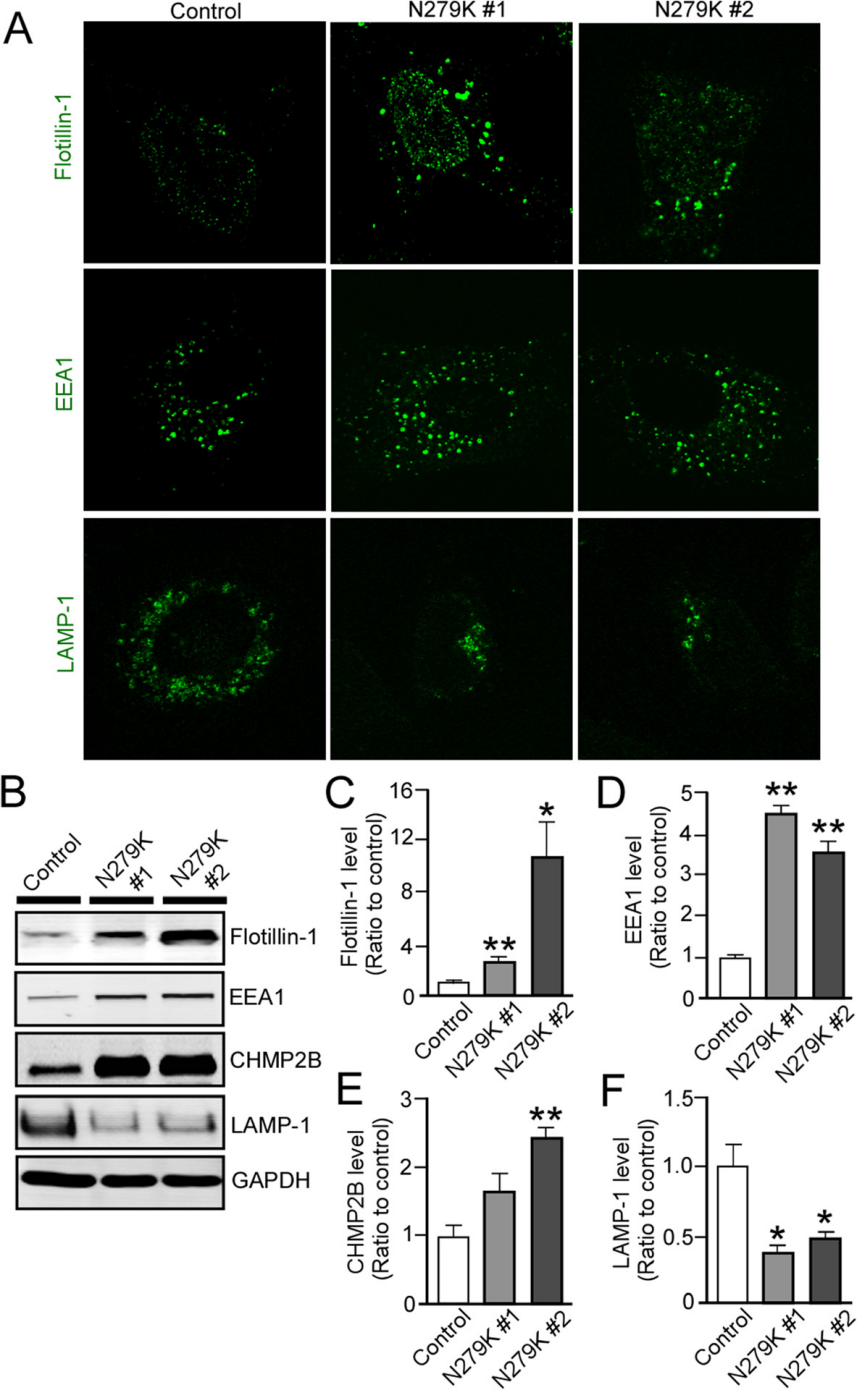
N279K tau mutant alters intracellular vesicle markers and their distributions in NSCs. **a** Immunostaining of Flotillin-1, EEA1 and LAMP-1 in control and N279K NSCs. **b**-**f** Western blot analysis of Flotillin-1, CHMP2B, EEA1 and LAMP-1 in control and N279K NSCs. Quantification of Western blot densitometry of Flotillin-1 **c**, EEA1 **d**, CHMP2B **e** and LAMP-1 levels **f**. Data are mean ± SEM from three independent experiments. *p < 0.05; **p < 0.01

**Fig. 4:**
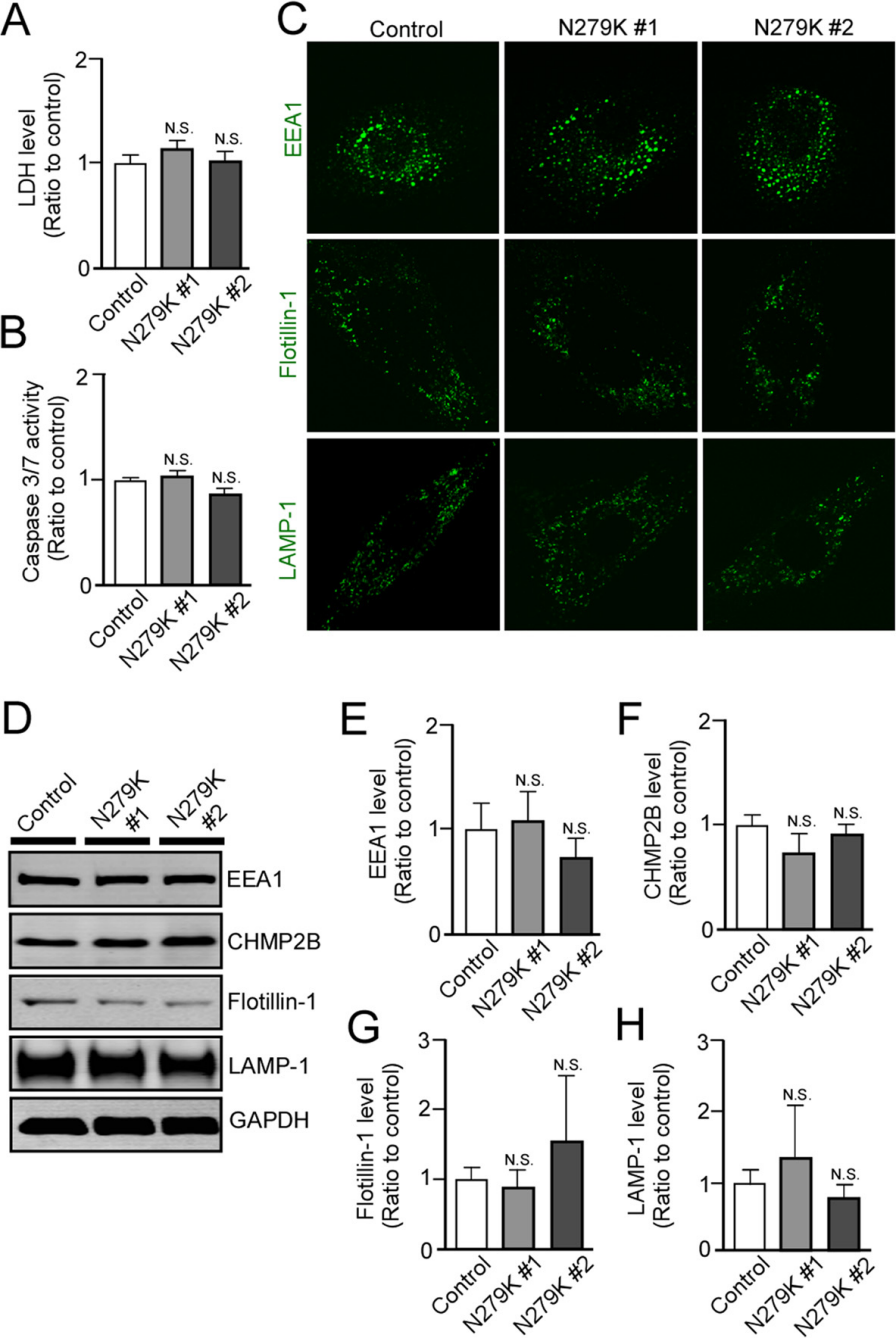
N279K fibroblasts do not display alterations in cellular stress or markers of vesicle trafficking. **a** LDH secretion was measured in control and N279K fibroblasts. The quantification was normalized to Bradford protein levels of cell lysate. **b** Caspase 3/7 activity was measured in control and N279K fibroblasts. The quantification was normalized to Bradford protein levels of cell lysate. **c** Immunostaining of control and N279K fibroblasts, demonstrating EEA1, Flotillin-1 and LAMP-1 positive punctates. **d**-**h** Expression levels of EEA1 **e**, CHMP2B **f**, Flotillin-1 **g** and LAMP-1 **h** in control and N279K fibroblasts were analyzed by Western blot. Data are mean ± SEM from three independent experiments. N.S., Not Significant

The physiological function of tau is to stabilize microtubules which are essential for proper intracellular trafficking [20]. Thus, to further explore N279K-mediated defects in NSCs, we probed for lipid raft marker Flotillin-1, which is indicative of intermediate intracellular vesicles including endosomes, multivesicular bodies and exosomes [21] (Fig. 3a). Control NSCs demonstrated the inferred basal expression of Flotillin-1, where the expression is sparse, punctate are few and are mainly localized in nuclear regions. Conversely, N279K NSCs displayed marked accumulation of Flotillin-1 signal in the form of small nuclear puncta and large vesicular puncta outside of the nuclear region (Fig. 3a). In addition, N279K NSCs expressed higher levels of the early endosome marker EEA1 observable as discrete puncta surrounding the nucleus as well as displayed denser assemblage of EEA1-reactive puncta compared to control NSCs (Fig. 3a). However, we found that lower immunoreactivity of a lysosome marker LAMP-1 in N279K NSCs than control NSCs. Correspondingly, Western blot showed that protein levels of Flotillin-1 (Fig. 3b, c) and EEA1 (Fig. 3b, d) were significantly increased in N279K NSCs than control NSCs. We also found increased levels of the charged multivesicular protein 2B (CHMP2B), which are indicative of higher levels of endosomal compartments chaperoned and matured to the late endosome stage in N279K NSCs than control NSCs (Fig. 3b, e). Consistent with the results from confocal microscopy, Western blot analysis demonstrated significantly depleted levels of LAMP-1 protein in N279K NSCs when compared to control NSCs (Fig. 3b, f). These results indicate that the N279K tau mutation causes the enlargement of intraluminal vesicle compartments including endosomes and exosomes, but not that of lysosomes. We did not observe any significant differences in immunostaining patterns (Fig. 4c) and protein levels for these markers between control and N279K skin fibroblasts (Fig. 4d-h), suggesting that the vesicle trafficking defects are specific to the neuronal lineage.

### Flotillin-1 levels are increased in the cortex of PPND/FTDP-17 patients with N279K tau mutation

**Fig. 5:**
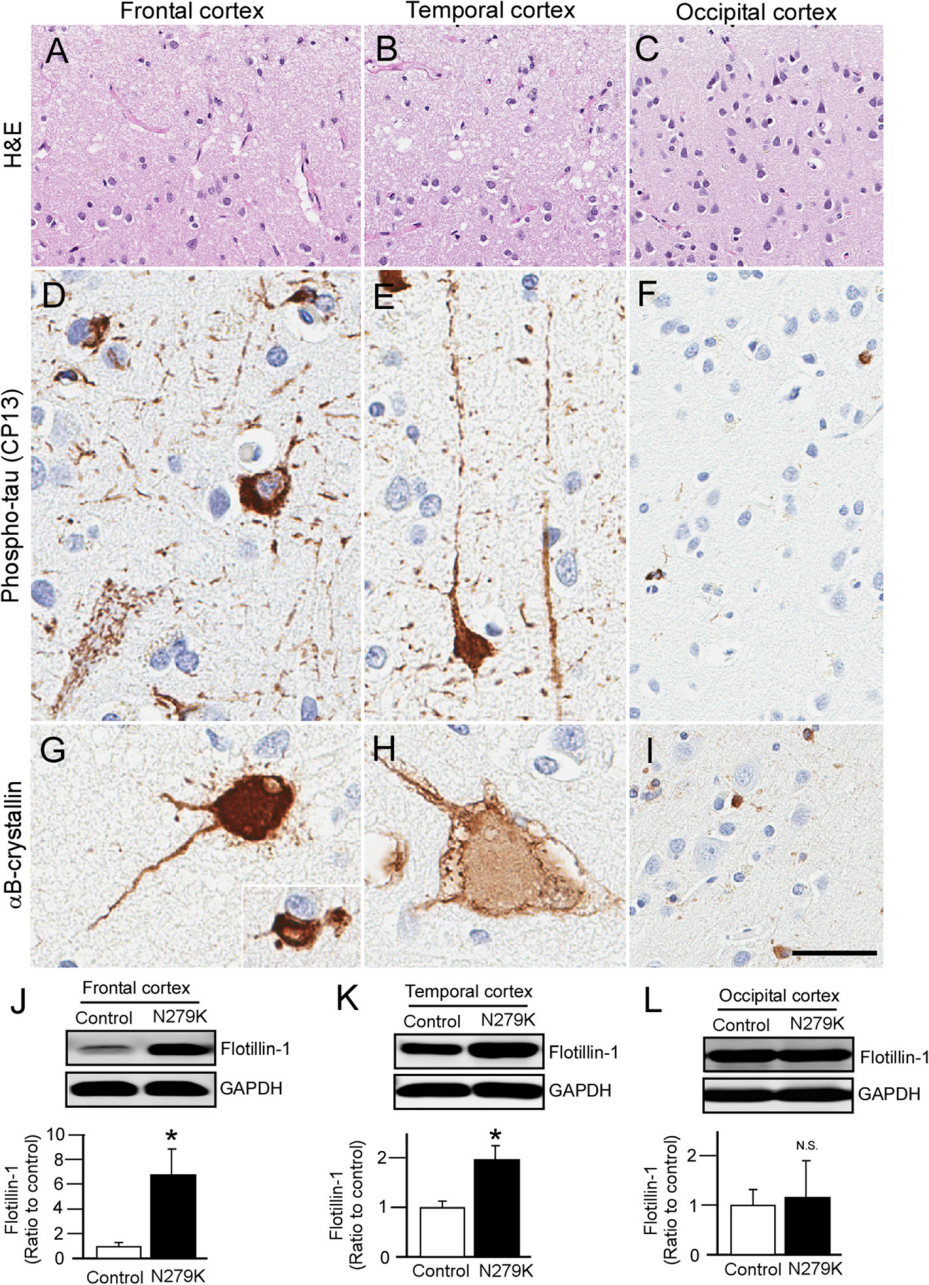
Histopathologic findings and Western blot analysis of cortex in autopsy-confirmed PPND/FTDP-17 patients with N279K tau mutant. **a** Neuronal loss and superficial microvacuolation demonstrated by H & E staining in the frontal and **b** temporal cortex, but **c** occipital cortex is relatively spared in PPND/FTDP-17 patients. Scale bar, 100 μm. **d** Phospho-tau-positive pre-tangles, threads, and coiled bodies in frontal cortex and **e** temporal cortex but, **f** only rare threads and glia are found in occipital cortex of PPND/FTDP-17 patients. Scale bar, 25 μm. **g** Ballooned neurons detected by αB-crystallin staining are found in the frontal and **h** temporal cortex, but **i** only rare αB-crystallin-positive glia are found in occipital cortex from PPND/FTDP-17 patients. Scale bar, 25 μm. **j**-**l** Western blot analysis of Flotillin-1 levels in the frontal **j**, temporal **k** and occipital **l** cortex from control non-demented controls (*n* = 4) and PPND/FTDP-17 patients (*n* = 4). Data are mean ± SEM. *p < 0.05

To investigate whether our findings obtained using patient-derived iPSCs occur in human brains, we analyzed four postmortem brains from PPND family members who were carriers of N279K tau mutation and affected by this illness. The median age at death was 52 years and median duration of illness was 6.6 years (Table 1). Neuronal loss and superficial microvacuolation were present in the frontal (Fig. 5a) and temporal cortices (Fig. 5b), but not evident in the occipital cortex – the most spared cortical region in PPND/FTDP-17 patients (Fig. 5c). The sections stained with a phospho-tau antibody reveals numerous tau-positive cell processes (“threads”), glia (astrocytes and oligodendroglial “coiled bodies”), and pre-tangles, in frontal (Fig. 5d) and temporal cortex (Fig. 5e), but only rare threads and coiled bodies in occipital cortex (Fig. 5f). αB-crystallin-positive balloon neurons and coiled bodies were found in frontal (Fig. 5g) and temporal cortex (Fig. 5h), but only rare labelling of glia were found in occipital cortex (Fig. 5i).

**Table 1:** Postmortem brain samples from PPND/FTDP-17 patients with N279K tau mutation

Age of Onset	Disease Duration	Age at Death	Gender	Race	Pathological Diagnosis	Mutation
-	-	61	Female	Caucasian	Normal	-
-	-	54	Female	Caucasian	Normal	-
-	-	56	Female	Caucasian	Normal	-
-	-	57	Female	Caucasian	Normal	-
44	6.5	50	Female	Caucasian	PPND/	*MAPT*
					FTDP-17	c.837 T > G (p.N279K)
43	8.0	51	Female	Caucasian	PPND/	*MAPT*
					FTDP-17	c.837 T > G (p.N279K)
48	4.8	53	Female	Caucasian	PPND/	*MAPT*
					FTDP-17	c.837 T > G (p.N279K)
46	6.8	53	Female	Caucasian	PPND/	*MAPT*
					FTDP-17	c.837 T > G (p.N279K)

**Fig. 6:**
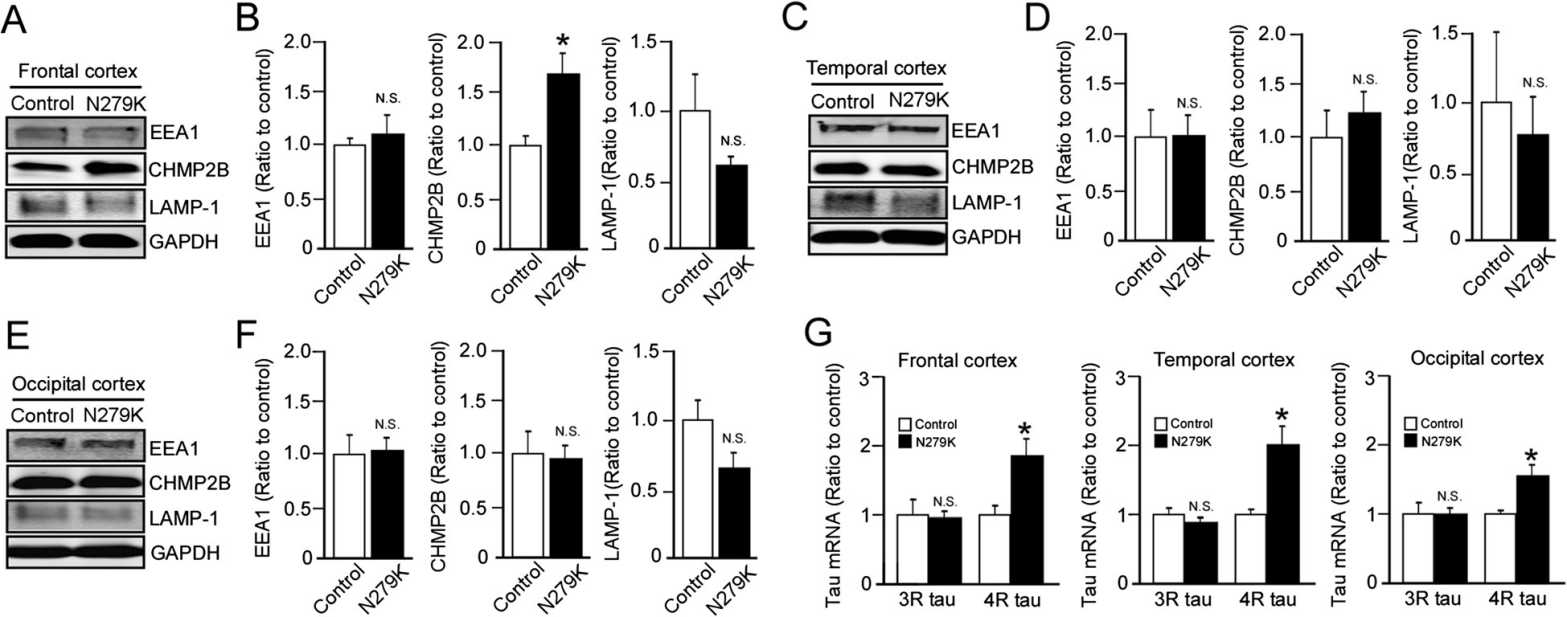
Subcellular vesicle components and tau mRNA levels in the cortices of PPND/FTDP-17 patients with N279K tau mutation. **a**-**f** Western blot analysis of EEA1, CHMP2B and LAMP-1 levels in the frontal **a**, **b**, temporal **c**, **d** and occipital **e**, **f** cortex from non-demented controls (*n* = 4) and PPND/FTDP-17 patients (*n* = 4). **g** Quantitative RT-PCR for levels of 3R-tau and 4R-tau mRNA in the frontal, temporal and occipital cortex from control non-demented controls (*n* = 4) and PPND/FTDP-17 patients (*n* = 4). Relative gene expression represents fold changes relative to control patient levels, normalized to β-actin. Data are mean ± SEM. *p < 0.05. N.S, Not Significant

We found that Flotillin-1 protein levels were significantly increased in the frontal (Fig. 5j) and temporal cortex (Fig. 5k) of PPND/FTDP-17 patients with N279K tau mutation compared to control individuals, which is consistent with our results from the iPSC-based analysis. In occipital cortex, no significant change was detected in Flotillin-1 levels between control individuals and the patients (Fig. 5l). Increased CHMP2B levels were also observed in the frontal cortex, but not in temporal and occipital cortices (Fig. 6a*-*f). In addition, there were no significant differences in EEA1 and LAMP1 levels in frontal, temporal and occipital cortex between and control and the N279K carriers (Fig. 6a*-*f). We also confirmed the up-regulation of 4R-tau mRNA levels in the cortex of these patients (Fig. 6g). Thus, these results indicate that NSCs derived from the patient-specific iPSCs model neuronal phenotypes of PPND/FTDP-17 patients with N279K tau mutation, where disturbances of intracellular vesicle trafficking in neuronal cells may contribute to the pathological changes.

## Discussion

Inheritance of one allele of *MAPT* with the N279K mutation is causal to the development of pre-geriatric dementia, in the form of FTDP-17 [14]. The mutation resides within an exonic splicing enhancer site, and increases the level of the isoform containing exon 10, resulting in enhanced 4R-tau mRNA production [22]. Whereas 4R-tau protein is shown to have a stronger interaction with microtubules than 3R-tau, 4R-tau is also more effective at assembling microtubules [23]; the N279K mutation does not appear to affect the binding of tau to microtubules or decrease the ability of tau to promote microtubule assembly when compared to wild-type 4R-tau [24]. Thus, it remains unclear how the N279K tau mutant leads to neurodegeneration and tauopathy in FTDP-17T patients. In this study, we demonstrate that NSCs carrying the N279K tau mutant exhibit higher levels of 4R-tau and markedly higher cellular stress compared with control NSCs using patient-specific iPSCs. Furthermore, we show that intracellular vesicle trafficking is significantly perturbed. Importantly, we show that N279K tau influences intracellular vesicle trafficking as early as the NSC stage, which could not be examined through previous *in vitro* experiments.

Endocytosed and intracellular products are packaged into membrane bound specialized vesicles that are trafficked along microtubules to reach their correct destination or perform their specialized function to ensure proper cell dynamics. Impairment in the orderly trafficking of proteins and lipids intracellularly has been documented to initiate and execute cell death processes. Furthermore, intra-organelle communication is also believed to play a crucial role in the initiation of cell death events [25]. Mounting evidence implicates trafficking alterations, in particular, de-regulation of the endosomal system in the pathogenesis of neurodegenerative diseases [26]. In this work, we find substantial increases in the levels of flotillin-1 in N279K NSCs. Flotillin proteins which represent scaffolds of lipid raft microdomains [21] are involved in the sorting of intracellular vesicle compartments toward Golgi apparatus, recycling endosomes, multivesicular bodies [27], late endosomes/lysosomes [28] and exosomes [29]. Thus, the accumulation of flotillin-1 in intracellular space is indicative of interruptions of proper vesicle trafficking or abnormal acceleration of endocytosis machinery. Furthermore, our results show markedly increased levels of an early endosome marker EEA1 and a late endosome marker CHMP2B in N279K NSCs. In contrast, N279K NSCs show marked depletion of lysosomal compartments and protein levels of lysosomal marker LAMP-1. Lysosomes are highly dynamic membrane bound organelles whose main role is to receive and terminally degrade components sequentially sent through the endocytic pathway and from autophagic processes [30]. Therefore, the possession of the tau N279K mutant may disrupt the delivery of waste products from endosomes to lysosomes in NSCs, resulting in enlarged intracellular vesicles such as endosomes and exosomes. The aberrant processing of intracellular vesicles eventually render NSCs more intrinsically vulnerable to internal stressors and initiate signaling of cell stress through their accumulation. This accumulation may saturate the endocytic pathway and lead to the theorized propagation of pathologic/harmful products through intercellular exosomal transfer. Whilst this is seen as a permissive and protective mechanism to relieve intracellular vesicular traffic, exosomal enrichment of pathogenic tau may be speculated as an underlying vector of disease spread in FTDP-17.

Consistent with our NSC data, we show the increased flotillin-1 in the frontal and temporal cortices in post-mortem brain tissue of N279K carriers, and increased CHMP2B levels in the frontal cortex of the PPND/FTDP-17 patients. Interestingly, mutations in *CHMP2B* are also causal for the development of FTD [31, 32] and amyotrophic lateral sclerosis [33]. CHMP2B is a subunit of endosomal sorting complex required for transport-III, which is involved in the degradation of proteins in the endocytic and autophagic pathways [34]. In fact, CHMP2B immunoreactive granules are observed as granulovacuolar degeneration in the brains of Parkinson’s disease, incidental Lewy body disease [35] and Alzheimer’s disease (AD) patients [36]. Flotillin-1 is also known to accumulate in tangle-bearing neurons in AD brains [37]. Thus, the alteration of vesicle trafficking is likely a common pathogenic event across a spectrum of neurodegenerative diseases, although further work is required to completely elucidate whether the trafficking disturbance is a cause or a consequence of neurodegeneration.

In FTDP-17T as well as other neurodegenerative diseases termed tauopathies, accumulation of tau pathology is the primary histological feature. Flotillin-1 is increased in the frontal and temporal cortex, but not in occipital cortex where tau accumulation and deposition is relatively mild. Since 4R-tau levels are increased in all three cortical regions from the patients, there might be specific molecular mechanisms to compensate for the harmful effects of increased 4R-tau due to N279K tau in occipital cortex. Mutant N279K tau may initiate or facilitate the aggregation of tau into filaments by affecting vesicle trafficking in NSCs and/or neurons later on in disease when compensatory mechanisms are expended. Exosomes provide a motile intercellular chamber for the delivery of endocytosed and intracellular molecules to the extracellular space, for the active uptake by neighboring cells, which has been recently hypothesized to be a major component in the phenomena of disease pathology propagation [38], especially involving tau [39]. Since flotillin-1 is abundantly localized and enriched in intraluminal vesicles/exosomes, it is possible that N279K tau mutation facilitates the formation of exosomes and potentiates the spreading of tau pathology, which exacerbates cellular viability later on in the course of the disease.

## Conclusions

Our results show that NSCs harboring the mutant N279K tau display altered levels of multiple intracellular vesicle trafficking markers, suggesting disruptions in the transport of membrane-bound organelles, which may ultimately lead to enhanced cellular stress observed in tau mutant cells. These findings have important implications for our understanding in the pathogenic mechanism of N279K tau mutation and those findings might be partly shared with sporadic FTD and/or PSP. Further studies are needed to elucidate the precise pathways by which N279K mutant tau alters intracellular vesicle trafficking. Importantly, our findings provide evidence that iPSC technology can be used to successfully model early dysfunctions in somatic cells derived from affected human patients, which can be complemented by post-mortem brain studies and may underlie future advances in the translatability of research involving PPND/FTDP-17 patients.

## Methods

### Isolation of primary human skin fibroblasts and generation of iPSCs

Studies were approved by Mayo Clinic under IRB protocols (09-003803 and 12-002562). Clinically affected two patients from PPND family were recruited; #1) 48 year-old patient (male, Caucasian) with the disease onset at 44 year-old and #2) 50 year-old patient (female, Caucasian) with the disease onset at 49 year-old. After informed consent was obtained, dermal skin biopsy specimens were harvested in a routine fashion from the medical aspect of non-dominant fore arm. The patients were clinically genetically tested to confirm N279K tau mutation status. DNA was extracted from the fibroblasts with standard protocol and Sanger sequencing for the c.837 T > G mutation (p.N279K) in exon 10 of the *MAPT* on chromosome 17 was performed using the primer pair 10 F (5’-TGC CTC TGC CAA GTC CGA AAG-3’) and 10R (5’-CCA GAT CCT GAG AGC CCA AGA AG-3’). Control fibroblasts were obtained from Cell Applications, Inc. (Catalog No. 106-5a; Lot No. 2023). Fibroblasts were amplified and maintained in fibroblast medium; DMEM culture medium (Invitrogen) containing 10 % fetal bovine serum (FBS) (Gemini Bio-Products), and supplemented with 1 % non-essential amino acids (NEAA) (Invitrogen), 1 % Penicillin-Streptomycin (Invitrogen), and 1 % Amphotecerin B (Gemini Bio-Products). The iPSCs were created by the electroporation of three episomal vectors containing, OCT3/4, SOX2, KLF4, L-MYC, LIN28, and p53-shRNA (Addgene), as described before [17], transfection was assisted with the NHDF nucleofection kit (Lonza). Cells were plated on a 35 mm dish in fibroblast medium, 24 h later, a 100 % media change was performed. Seven days after nucleofection, cells were trypsinzed (0.25 % trypsin/1 mM EDTA, Gibco) and re-plated onto a MEF feeder layer (Global Stem) in a 100 mm dish in fibroblast medium. After 24 h, medium was replaced with mTeSR1 complete medium (STEMCELL Technologies) and was changed every day. The iPSC colonies were isolated and expanded approximately after 3-4 weeks in culture. The iPSC colonies were passaged using dispase (STEMCELL Technologies) and subject to treatment with rock inhibitor Y27632 (Sigma-Aldrich).

### Differentiation of iPSCs

Embryonic bodies were produced after treating iPSC colonies with dispase and placing them in human neural stem cell differentiate medium (50 % DMEM/F12 plus 50 % Neural basal medium (Gibco) containing 1 % N2 supplement (Gibco), 2 % B27 supplement (Gibco), 1 μM Dorsomorphin (Sigma-Aldrich), 10 μM SB431542 (Sigma-Aldrich)) in a 100 mm petri dish. Medium was changed every two days. After 2 weeks, spheres were seeded on polyornithine (Sigma-Aldrich) and laminin (Sigma-Aldrich) coated dishes and kept culturing in the same medium for another 7-14 days for neural rosette formation. Neural rosettes were collected with Stem Diff neural rosette selection reagent and re-plated onto polyornithine/laminin coated dishes as a single cell suspension in neural stem cell culture medium (ReNcell NSC maintenance media (Millipore) containing, 1 % N2 supplement, 1 % NEAA, 20 ng/ml bFGF and 20 ng/ml EGF). For three germ layers differentiation, iPSC colonies were digested with dispase and culture in DMEM with 10 % FBS for 4 days in 10 cm petri dish. After 4 days in suspension culture, floating embryonic bodies were re-seeded onto gelatin-coated dishes in the same culture medium for 10 days. The medium was changed every other day.

### Immunocytochemistry

Cells were passaged on 4 well slides (Lab-Tek 0.1 borosilicate glass slides) at 37 °C before experiments. Once iPSC colonies grew large enough or reached final differentiation stage, cells were briefly fixed in 4 % paraformaldehyde and then permeabilized in 0.2 % Triton X-100 in PBS. Cells were blocked with 10 % normal goat serum for 30 min, before incubation with primary antibody for 1 h at room temperature. Cells were incubated with primary antibodies against Nanog (Cell Signaling), TRA-1-60 (Chemicon International), TRA-1-81 (Chemicon International), AFP (R & D Systems), SMA (Abcam), Tuj1 (Covance), Synaptophysin (Millipore), Pax6 (Abcam), Nestin (Abcam), Flotillin-1 (Novus Biologicals), Lamp1 (Santa-Cruz), G3BP (BD Biosciences), TIA-1 (Santa-Cruz) or EEA1 (BD Transduction Laboratories). After washing with PBS, cells were incubated with Alexa Fluor-488 or 568 conjugated secondary antibodies for 1 h at room temperature. Fluorescent signals were detected by fluorescence microscopy (model IX71 Invert, Olympus) or confocal laser scanning fluorescent microscopy (model LSM510 Invert, Carl Zeiss) and images were processed using Photoshop.

### RNA extraction and RT-PCR

Total cellular RNA was extracted from cells using RNeasy mini kit (Qiagen), according to the manufacturer’s instructions, and subject to DNase 1 digestion to remove contaminating genomic DNA. Total RNA was dissolved in nuclease-free water and reverse transcribed using the Superscript III First-Strand synthesis System (Invitrogen). The cDNA from the reaction mix was subject to quantitative real-time PCR to detect levels of each corresponding gene. The set of actin primers was used as an internal control for each specific gene amplification. The relative levels of gene expression were quantified through use of the Bio-Rad iCycler iQ software. The primers used to amplify target genes by RT-PCR and quantitative PCR were as follows; endogenous hOCT3/4 F (5’-GAC AGG GGG AGG GGA GGA GCT AGG-3’) and R (5’-CTT CCC TCC AAC CAG TTG CCC CAA AC-3’), endogenous hSOX2 F (5’-GGG AAA TGG GAG GGG TGC AAA AGA GG-3’) and R (5’-TTG CGT GAG TGT GGA TGG GAT TGG TG-3’), endogenous hKLF4 F (5’-ACG ATC GTG GCC CCG GAA AAG GAC C-3’) and R (5’-TGA TTG TAG TGC TTT CTG GCT GGG CTC C-3’), endogenous hc-MYC F (5’-GCG TCC TGG GAA GGG AGA TCC GGA GC-3’) and R (5’-TTG AGG GGC ATC GTC GCG GGA GGC TG CAG-3’), GDF3 F (5’-CTT ATG CTA CGT AAA GGA GCT GGG-3’) and R (5’-GTG CCA ACC CAG GTC CCG GAA GTT-3’), REX1 F (5’-CAG ATC CTA AAC AGC TCG CAG AAT-3’) and R (5’-GCG TAC GCA AAT TAA AGT CCA GA-3’), NANOG F (5’-CCC CGA TTC TTC CAC CAG TCC C-3’) and R (5’-CGG AAG ATT CCC AGT CGG GTT CAC C-3’), FGF4 F (5’-CTA CAA CGC CTA CGA GTC CTA CA-3’) and R (5’-GTT GCA CCA GAA AAG TCA GAG TTG-3’), ESG1 F (5’-ATA TCC CGC CGT GGG TGA AAG TTC-3’) and R (5’-ACT CAG CCA TGG ACT GGA GCA TCC-3’), 3R-tau F (5’-TTG CTC AGG TCA ACT GGT TTG TA-3’) and R (5’-ACT GAG AAC CTG AAG CAC CA-3’), 4R-tau F (5’-GAA GCT GGA TCT TAG CAA CG-3’) and R (5’-TTA CTT CCA CCT GGC CAC CTC CT-3’), β-actin F (5’-CTG GCA CCA CAC CTT CTA CAA TG-3’) and R (5’-AAT GTC ACG CAC GAT TTC CCG C-3’).

### Cell toxicity assays

Caspase 3/7 activity was assessed using a Caspase-Glo 3/7 assay (Promega) according to the manufacturer’s directions. Background readings were subtracted from the measured luminescence value and positive controls were present in each assay and cell type using 0.5 μM of staurosporine treatment for 18 h, to ensure signal validity. LDH concentration was measured in cell culture supernatant that had been pre-incubated on confluent cells for 24 h, and was detected using the LDH activity assay kit (Sigma), according to manufacturer’s instructions.

### Western blotting

Cells were lysed in 1 % Triton X-100 PBS containing 1 % protease inhibitor cocktail (Roche), triturated and centrifuged at 13,000 g for 10 min at 4 °C. Brain lysates were prepared from frozen brain tissue using a one-step extraction method, where 100 mg of tissue was lysed in ice-cold 1 % Triton X-100 PBS containing 1 % protease inhibitor cocktail and centrifuged at 100,000 g for 15 min at 4 °C. Samples were separated on 10 % SDS-polyacrylamide gels and transferred to PVDF Immobilon FL membranes (Millipore) overnight. Membranes were blotted with primary antibodies against Flotillin-1 (Novus Biologicals), Lamp-1 (Santa-Cruz), G3BP (BD Biosciences), TIA-1 (Santa-Cruz), EEA1 (BD Transduction Laboratories) or CHMP2B (Abcam) in 5 % non-fat milk containing 0.01 % Tween-20. Odyssey IR680 or IR800 secondary antibodies against the species of the primary antibody were incubated for 1 h at room temperature. Immunoreactive bands were detected and quantified using the odyssey infrared imaging system (LI-COR Biosciences).

### Human brain sample analysis

Postmortem tissues were obtained through the Mayo Clinic Jacksonville brain bank for neurodegenerative diseases. All brains were donated from affected PPND family members who were clinically genetically confirmed to be N279K tau mutation carriers. Demographic characteristics are shown in Table 1. A standardized detailed neuropathologic assessment included routine histological examination with hematoxylin & eosin (H & E) staining as well as immunostaining with a phospho-tau monoclonal antibody phospho-tau antibody (CP13, mouse IgG1, 1:1,000, kind gift of Peter Davies, Feinstein Institute for Medical Research, North Shore LIJ Health Care System) and αB-crystallin (Rabbit polyclonal, 1/5,000 dilution; Chemicon, Temecula, CA).

### Statistical analysis

All quantified data represents an average of samples. Statistical significance was determined by two-tailed paired student’s *t* test, and *p* < 0.05 was considered significant.

## Abbreviations

AD: Alzheimer’s disease
AFP: α-fetoprotein
CHMP2B: Charged multivesicular protein 2B
FTDP-17: Frontotemporal dementia with parkinsonism related to chromosome 17
GRN: Progranulin
iPSCs: Induced pluripotent stem cells
LDH: Lactate dehydrogenase
MAPT: Microtubule-associated protein tau
NSCs: Neural stem cells
PPND: Pallido-ponto-nigral degeneration
SMA: α-smooth muscle actin
